# Type I allergic reaction to rituximab upon first lifetime exposure: a case report

**DOI:** 10.1186/s13223-020-00448-8

**Published:** 2020-06-26

**Authors:** V. Polito, A. Barbacki, G. Isabwe

**Affiliations:** 1 McGill University, Montreal, QC Canada; 2 Division of Allergy and Clinical Immunology, Department of Medicine, McGill University Health Centre, McGill University, Montreal, QC Canada

**Keywords:** Rituximab, Monoclonal antibody, MAB, Hypersensitivity, IgE, Anaphylaxis, Drug allergy, Infusion reaction, Cytokine reaction, Type I reaction

## Abstract

**Background:**

While drug reactions to rituximab have been commonly reported in the literature, a type I allergic reaction to rituximab after first lifetime exposure has never been reported.

**Case presentation:**

We describe a case of a 58-year-old female patient who received rituximab for the first time for treatment of rheumatoid arthritis. She developed symptoms immediately after infusion, however presented 11 days after drug exposure with cyclical anaphylaxis-like reaction requiring multiple doses of epinephrine. On second exposure, she experienced immediate anaphylaxis 30 min into infusion.

**Conclusion:**

Our case illustrates the importance of heightened awareness by physicians that type I IgE-mediated reactions after first exposure to monoclonal antibodies such as rituximab are possible, and if unrecognized, could be potentially life-threatening.

## Background

Rituximab is a chimeric monoclonal antibody (MAB), which is approximately 65% human, the remainder being mouse epitope. It binds to the CD20 antigen on B cells and is primarily used in the treatment of autoimmune disorders and malignancies [1]. We describe an unusual presentation of rituximab hypersensitivity.

## Case presentation

Our patient is a 58-year-old woman known for rheumatoid arthritis, depression, and migraines. Her medications include bupropion and low-dose prednisone (5–10 mg [mg] daily). She has no personal or family history of allergy or urticaria. She previously failed treatment for her rheumatoid arthritis with methotrexate, tocilizumab, and tofacitinib. During her first infusion of rituximab (administered over 4 h), she developed fatigue and a migraine which persisted for 4 days post-infusion. On day 2 post-rituximab, she also developed 1 day of throat pain. On day 10, the patient had transient diffuse scalp pruritus. On day 11, she developed pruritus which developed into urticaria followed by face and tongue angioedema and throat tightening. On presentation to the emergency room (ER), she was tachycardic (at 123) with otherwise normal vital signs and normal physical exam. She was given famotidine 20 mg and methylprednisolone 80 mg intravenously (IV), and diphenhydramine 50 mg orally (PO). Despite initial improvement of her symptoms, the patient’s urticaria, angioedema, and chest tightness with wheezing re-occurred. She was given a dose of epinephrine 0.5 mg IM. The patient remained in the ER for over 48 h with recurrences of her symptoms necessitating IM epinephrine a total of three times. Repeated vital signs were normal other than intermittent tachycardia (100–125). Bloodwork showed a C-reactive protein (CRP) of 144.14 mg/L (liter), a tryptase of 11.9 μg (microgram)/L done 15 h after arrival, and a white blood cell count of 15.60. Once stable, she was discharged home with cetirizine 10 mg PO daily as needed.

Twenty-four hours after discharge, the patient returned with subjective symptoms of pruritus and body aches. She had received epinephrine IM in ambulance. Bloodwork showed a CRP of 52.96 mg/L and a tryptase of 3.4 μg/L. There was no objective evidence of ongoing reaction. She was discharged home with a PO prednisone taper. She was subsequently seen in the allergy clinic and at that time, the reaction was thought to be unlikely secondary to Rituximab.

On day 26, she received her second dose of Rituximab in an outpatient clinic. She was pre-medicated with acetaminophen 650 mg PO, diphenhydramine 50 mg IV and methylprednisolone 125 mg IV. Thirty minutes after initiation of the infusion, the patient developed symptoms of chest pain and throat tightness. Objectively, she was found to have urticaria at the infusion site and became hypotensive with a systolic blood pressure of 94 from 140, along with hypoxia requiring 5 L/min oxygen via nasal prong to maintain a saturation of 95%. The rest of her vital signs were normal. She was given epinephrine IM as well as diphenhydramine 25 mg IV and transferred to ER. In the ambulance, her oxygen requirement and hypotension quickly resolved post-administration of epinephrine. Upon arrival she was asymptomatic, with normal vital signs. Her CRP was 3.11 mg/L and tryptase was 5.2 μg/L. After observation for 12 h, she was discharged on PO prednisone with a slower taper to home dose, and was referred back to the allergy clinic.

Rituximab skin prick test was negative at a concentration of 10 mg/mL. Intradermal skin testing was started at 1:1000 dilution (0.01 mg/mL) and quickly became positive with a wheal of 6 mm and flare of 20 mm. Saline control was negative. Histamine control showed a wheal of 5 mm.

## Discussion and conclusion

Reports of mild infusion reactions with rituximab are common, particularly during first infusions. Molecular studies seem to suggest a key role for complement system activation for infusion reaction [2]. Common symptoms include flushing, fever, rigors, and malaise. They improve with symptomatic treatment (anti-pyretic, anti-emetic medications, steroids) or slower infusion, and tend not to recur. Cytokine-mediated reactions can present with similar symptoms, however unlike infusion reactions, they can persist many days post-infusion, do not respond to symptomatic treatment, and will recur with subsequent infusions [3, 4]. Type I reactions to Rituximab are frequent and both IgE and non-IgE mediated. Symptoms may involve multiple organ systems. Skin-testing to Rituximab is one way to help confirm IgE-mediated allergy [5, 6].

In this case, since the patient’s symptoms persisted more than 24 h post-infusion, we question a potential cytokine-release reaction initially. On day 11 after initial exposure to rituximab, the patient presented with urticaria and angioedema which can be consistent with Type I reaction versus other unrelated condition such as spontaneous urticaria/angioedema. However, we hypothesize that, given the half-life of rituximab in the blood for rheumatoid arthritis is approximately 18–23 days [7], circulating antigens in the bloodstream caused the patient to develop antibodies to rituximab and convert from a cytokine-mediated reaction to a Type I reaction. In retrospect, this theory was supported by her elevated tryptase level of 11.9 μg/L (from baseline tryptase of 3.4 μg/L, using formula 1.2 × baseline tryptase + 2 μg/L) [8]. The persistence of circulating antigens in her bloodstream may also explain why the patient had a cyclical nature to her symptoms requiring multiple doses of epinephrine. Given it was the first exposure to rituximab, and given alternative possible explanations to her first presentation (for example, spontaneous urticaria/angioedema), the allergy clinic initially thought her first reaction was unrelated to rituximab. However, her symptoms after second exposure along with subsequent evidence of positive skin testing confirmed a true type I hypersensitivity to rituximab. As well, there was no history and no recurrence of urticaria/angioedema outside of exposure to rituximab.

Another potential question may be cross-reactivity between rituximab and the patient’s previous MAB treatment with tocilizumab due to the mouse epitope in both. We do not think this was the case since the patient received doses of tocilizumab on multiple occasions in the past without any reaction. The literature also supports that cross-reactivity is unlikely, given that there are no reported cases of proven anaphylaxis after first exposure due to cross-reactivity between MABs [9]. In fact, there are reports of safe administration of rituximab after anaphylaxis to obinutuzumab, which belongs to the same class of MAB as tocilizumab and is over 95% humanized [1, 10].

We have described an unusual case of cytokine-mediated reaction to rituximab after a first infusion, which subsequently converted to a type I IgE-mediated reaction after only one infusion of rituximab. To our knowledge, this is the first reported case of this type of reaction to rituximab. The patient and her treating team were made aware that desensitization for future infusions could be pursued, however, they decided to opt for an alternative treatment regimen.

## Data Availability

Not applicable.

## Abbreviations

MAB: Monoclonal antibody
mg: Milligram
IM: Intramuscularly
IV: Intravenously
PO: Orally
CRP: C-reactive protein
L: Liter
μg: Microgram
ER: Emergency room
